# Mitochondrial dysfunction-targeting therapeutics of natural products in Parkinson’s disease

**DOI:** 10.3389/fphar.2023.1117337

**Published:** 2023-05-10

**Authors:** Ting He, Xiaoyan Lin, Anping Su, Yujie Zhang, Zhichao Xing, Li Mi, Tao Wei, Zhihui Li, Wenshuang Wu

**Affiliations:** Division of Thyroid Surgery, Department of General Surgery and Laboratory of Thyroid and Parathyroid Disease, Frontiers Science Center for Disease-related Molecular Network, West China Hospital, Sichuan University, Chengdu, China

**Keywords:** Parkinson’s disease, natural products, mitochondrial dysfunction, pharmacological mechanisms, flavanoids, phenols

## Abstract

Parkinson’s disease (PD), the second most common neurodegenerative disease worldwide, often occurs in middle-aged and elderly individuals. The pathogenesis of PD is complex and includes mitochondrial dysfunction, and oxidative stress. Recently, natural products with multiple structures and their bioactive components have become one of the most important resources for small molecule PD drug research targeting mitochondrial dysfunction. Multiple lines of studies have proven that natural products display ameliorative benefits in PD treatment by regulating mitochondrial dysfunction. Therefore, a comprehensive search of recent published articles between 2012 and 2022 in PubMed, Web of Science, Elesvier, Wliey and Springer was carried out, focusing on original publications related to natural products against PD by restoring mitochondrial dysfunction. This paper presented the mechanisms of various kinds of natural products on PD-related mitochondrial dysfunction regulation and provided evidence that natural products are promising to be developed as drugs for PD therapeutics.

## 1 Introduction

Parkinson’s disease (PD) is a progressive debilitating neurodegenerative disease worldwide that affects approximately 0.1%–0.2% of the general population but approximately 1% of the population over the age of 60 years in modern countries (Wright Willis et al., 2010; Zou et al., 2014). As an age-related neurodegenerative disease, PD onset has shown a serious increase in prevalence rates in the past 60 years (Muhammad et al., 2022). Due to global population aging, the prevalence of PD is expected to increase, and therapies for this disease may face large challenges in the future. The main clinical manifestations of PD are bradykinesia, tremor, postural instability, cognitive impairment and other nonmotor impairments (Blauwendraat et al., 2020; Mohammadipour et al., 2020), and the main pathological features are progressive loss of dopaminergic neurons in the substantia nigra striatum, deposition of neuronal α-synaptic nucleoproteins and formation of Lewy bodies with complex pathogenesis, including mitochondrial dysfunction, oxidative stress, neuroinflammation and so on (Kalia and Lang, 2015). Various treatment strategies have been put into effect, for example, clinical treatment with pharmacotherapy such as levodopa, dopamine agonists and anticholinergics, as well as nondrug interventions such as surgery and exercise (Voon et al., 2017). However, long-term use of these treatments can lead to serious side effects but fail to completely halt disease progression. Therefore, it is of great significance to develop novel drugs that are safe and effective for PD prevention and treatment.

Mitochondria are vital organelles that provide energy to cells and play a particularly important role as the cellular “powerhouse” of dopaminergic neurons (Schapira and Patel, 2014; Gao et al., 2022). Increasing evidence supports the critical role of mitochondrial dysfunction, such as adenosine triphosphate (ATP) depletion, oxidative stress elevation, aberrant mitochondria-dependent apoptosis and other effects, in the development of PD (Cong et al., 2016; Shahba et al., 2021), suggesting that targeting mitochondrial dysfunction is a promising therapeutic target for PD treatments. Natural products originated from plants, animals, or other natural sources have been applied as substances with therapeutic potential to treat different diseases. As reported in previous studies, many kinds of natural products, such as flavonoids and polyphenols, have manifested the strong effects of PD treatment by targeting mitochondrial dysfunction (Kim et al., 2017; van der Merwe et al., 2017). Therefore, this paper provides a brief review of the roles that various natural products play by targeting mitochondrial dysfunction in PD, highlighting the mechanisms of effects to shed light on developing novel therapeutics for PD.

## 2 Mitochondrial dysfunction and PD

Mitochondria are the energy suppliers of the cells. Mitochondrial respiration can support a vast amount of energy in the ATP form by the oxidative phosphorylation cycle and regulate energy-dependent cell functions, including intermediary metabolism, protein folding, and cell motility (Wallace et al., 1999; Jin et al., 2021). In addition, mitochondria are dynamic organelles that undergo constant fusion and fission (Ashrafi and Schwarz, 2013). Mitophagy is the selective clearance of damaged mitochondria via autophagy. Mitochondria strive to maintain a balance between biogenesis and mitophagy to maintain mitochondrial quality (Ventura-Clapier et al., 2008; Voigt et al., 2016; Wang et al., 2021). In addition, reactive oxygen species (ROS), which are intimately related to oxidative stress, can be generated through the electron carriers of the respiratory chain (Wang et al., 2015). ROS cause mitochondrial membrane lipid peroxidation and protein nitrification; damage mitochondrial DNA, mitochondrial membrane structure and electron transport chain enzyme complexes; weaken or prevent oxidative phosphorylation; reduce ATP production; release caspase-3; and trigger further mitochondrial damage, which leads to mitochondrial dysfunction (Ali et al., 2015; Badshah et al., 2016).

**FIGURE 1:**
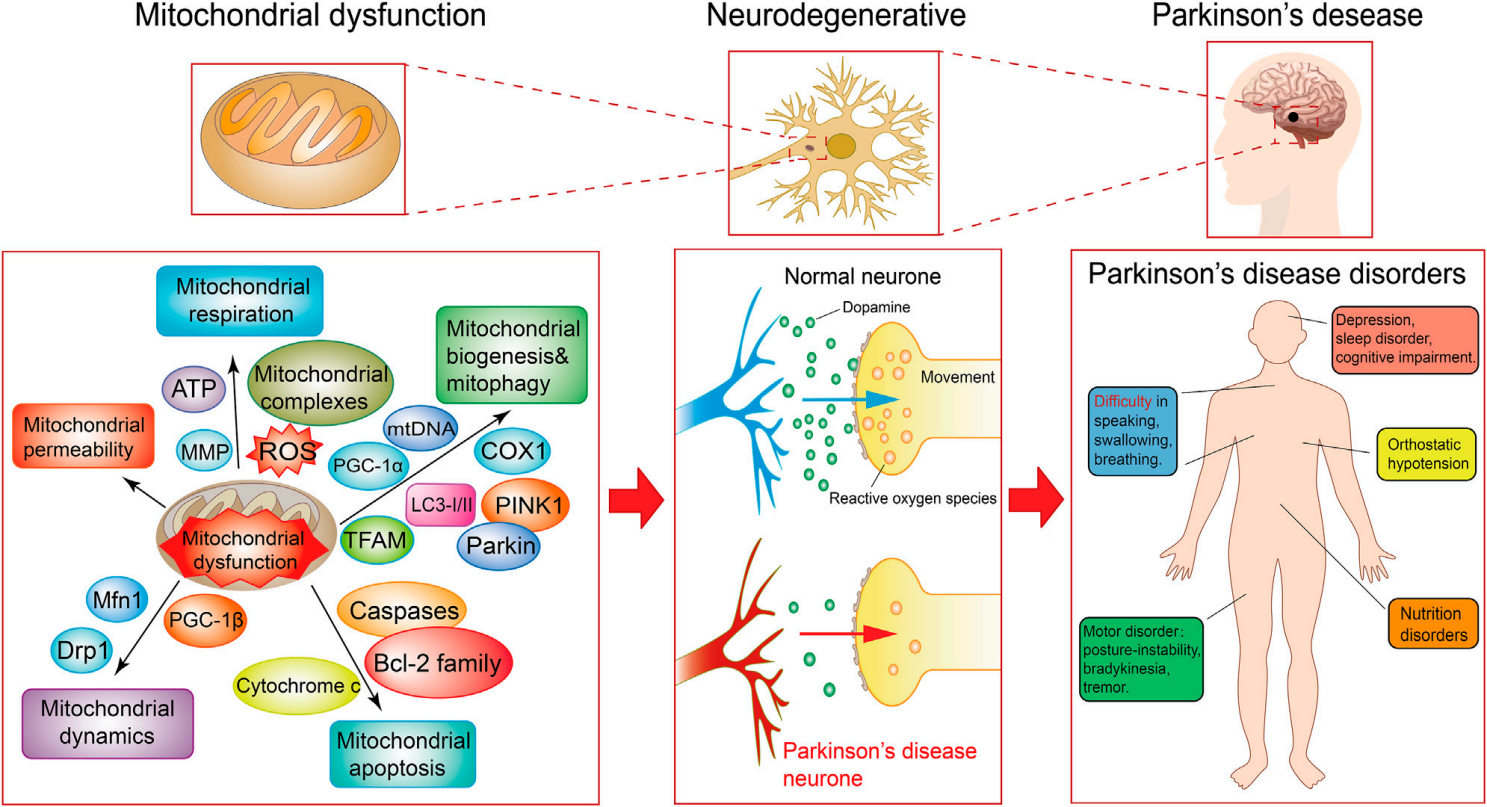
Primary mitochondrial dysfunction associated pathways and disease progression in Parkinson’s disease. Mechanisms of mitochondrial dysfunction in Parkinson’s disease include dysregulation of mitochondrial respiration, apoptosis, dynamics, biogenesis and mitophagy related to a variety of molecules. Mitochondrial dysfunction resulted in progressive loss of dopaminergic neurons in the substantia nigra striatum and ultimately cause severe motor and nonmotor disorders in Parkinson’s disease.

A number of studies have shown that dysfunction of related mitochondrial respiration is of great importance in PD progression (Figure 1). Neurons have a higher energy demand to maintain their function. ATP deficiency induced by mitochondrial dysfunction leads to a decrease in vesicular dopamine uptake, resulting in increased dopaminergic degradation in PD neurons (Wu et al., 2021). ROS, which are a prime culprit for causing mitochondrial dysfunction, play a pivotal role in the pathogenesis of PD (Bhattacharjee and Borah, 2016; Lu et al., 2016). In previous studies, it was also reported that impaired mitochondrial biogenesis led to depletion of mitochondria and that the imbalance of biogenesis and mitophagy is a mechanism mediating PD (Zhu et al., 2012; Liu et al., 2021a). Mitochondrial fission is a process of mitochondrial dynamics. When equilibrium is disrupted, excessive mitochondrial fragmentation leads to mitochondrial dysfunction and neuronal death in PD (Wang et al., 2022). In addition, mitochondrial apoptosis has also been reported in many PD studies (Khan et al., 2018; Balakrishnan et al., 2021). Thus, it is obvious that neurons are highly sensitive to mitochondrial dysfunction in PD and that mitochondria may be a target with great potential.

## 3 Natural products targeting mitochondrial dysfunction in PD

**FIGURE 2:**
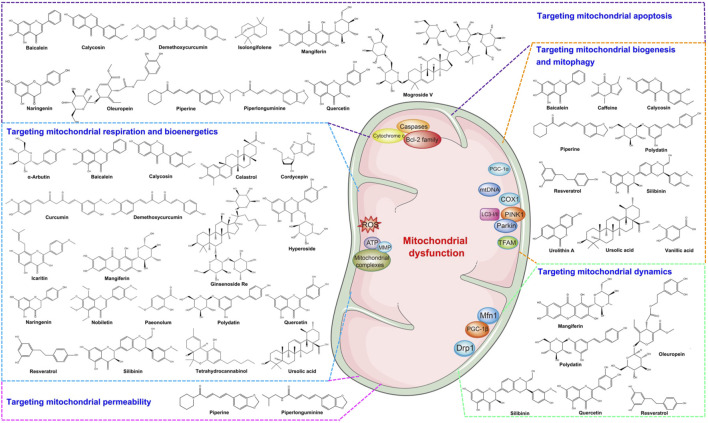
The structure of natural products targeting therapeutics in PD.

Natural products have been shown to have therapeutic potential for various diseases. Many kinds of natural products, such as flavonoids, polyphenols, terpenoids, and glycosides, have strong effects on PD by targeting mitochondrial dysfunction (Figure 2). Then, we reviewed the natural compounds and their molecular mechanisms by targeting mitochondrial dysfunction from different mechanisms of action (Table 1).

**TABLE 1:** Natural compounds targeting to mitochondrial dysfunction and PD models.

Category	Compounds or extracts	Biological source	*In vitro* experiments			*In vivo* experiments			Mechanisms				Refs
			Models	Dosage	Time	Models	Dosage	Time	Targeting mitochondrial respiration and bioenergetics	Targeting mitochondrial biogenesis and mitophagy	Targeting mitochondrial dynamics	Targeting mitochondrial apoptosis	
Flavanoids	Baicalein	*Scutellaria baicalensis*	Rotenone-induced SH-SY5Y cells	10 µM	24 h	Rotenone-treated mice	100 mg/kg	6 weeks	MMP↑	LC3B-II↑, autophagic flux↑	-	Caspase-3 activity↓	Kuang et al. (2017)
	Calycosin	*Astragalus membranaceus*	-	-	-	Paraquat-exposed drosophilas	0–400 μM	5 days	ATP, MMP, complex I and III activity↑	p62, phosphorylation levels of S6K and 4EBP1↓	-	JNK phosphorylation and caspase-3 activation↓	Chaouhan et al. (2022)
	Hyperoside	*Acer tegmentosum*	6-OHDA-induced SH-SY5Y cells	0.1–2 μM	24 h	-	-	-	MMP↑; ROS↓	-	-	-	Gao et al. (2022)
	Icaritin	*Epimedium sagittatum maxim*	-	-	-	MPTP-injected mice	4.7–18.9 mg/kg	5 days	ATP, ADP, inosine, and citric acid↑	-	-	-	Wu et al. (2021)
									SDHA, VDAC and ATP5B↑				
	Myricitrin	*Myrica cerifera*	MGO-induced SH-SY5Y cells	0.1–10 μM	24 h	-	-	-	ATP, MMP↑; ROS↓	-	-	-	Wang et al. (2014)
									AGEs/RAGE/NF-κB pathway↓				
	Naringenin	*Citrus reticulata*	MG-induced SH-SY5Y cells; Paraquat-induced SH-SY5Y cells	10–100 μM	2/24 h	Paraquat -exposed rats	40 mg/kg	21 days	MMP, ATP, complex I and V activity↑	-	-	Bcl-2↑; Bax, Cyt-c release, caspase-3/9 activation↓	de Oliveira et al. (2019), Ahmad et al. (2021)
	Nobiletin	*Citrus depressa* or *Citrus sinensis*	Lipopolysaccharide-exposed BV-2 cells	0–100 μM	4 h	Lipopolysaccharide-exposed mice	100 mg/kg	6 weeks	MMP, complex I-IV↑; ROS↓	-	-	-	Qi et al. (2019)
	Quercetin	*Bupleurum chinense* or *Crataegus pinnatifida*	-	-	-	Aluminumtreated rats	10 mg/kg	12 weeks	ROS↓; superoxide dismutase activity↑	-	Improving the mitochondrial integrity	Bax/Bcl-2 ratio, Cyt-c release, caspase-3↓	Sharma et al. (2016)
	Silibinin	*Silybum marianum* (L.) *Gaertn*	-	-	-	MPTP (1-methyl-4-phenyl-1,2,3,6-tetrahydropyridine)- induced mice	70, 140 and 280 mg/kg	24 days	MMP↑	PINK1, Parkin↑	Mfn1↑; Drp1↓	-	Liu et al. (2021a), Liu et al. (2021b)
Phenols and polyphenols	Curcumin	*Curcuma longa* (turmeric)	Paraquat -induced SH-SY5Y cells and PINK1 siRNA transfected SH-SY5Y cells	2 μM	1 h	Rotenone-treated mice	50, 100 and 200 mg/kg	21 days	MMP, complex II and IV activity, maximal respiration↑	-	-	-	Khatri and Juvekar (2016), van der Merwe et al. (2017)
	Demethoxycurcumin	A derivative of curcumin	Rotenone-induced SH-SY5Y cells	0–1 μM	28 h			-	MMP↑; ROS↓	-	-	Bcl-2, Bcl-xL↑; Bax, Bad, caspase-3/6/8/9, Cyt-c release↓	Ramkumar et al. (2017)
	Green tea polyphenols	-	Glutamate-treated primary cortical neurons	0.5–10 μM	24 h	-	-	-	-	-		Bcl-2↑; Bax, caspase-3↓	Cong et al. (2016)
	Mangiferin	*Mangifera indica*	Rotenone-induced SK-N-SH cells	2.5, 5, 10, 20 and 40 μg/mL	4 h	MPTP-induced mice	10, 20 and 40 mg/kg	12 days	ATP, MMP↑	-	Improving the mitochondrial integrity	Cyt-c release, caspase-3/9↓	Kavitha et al. (2014), Wang et al. (2022)
	Oleuropein	*Fraxinus rhynchophylla*	Glutamate-treated HT-22 cells	0–20 μM	1 h	-	-	-	ATP↑; ROS↓	-	Drp1↓	Bcl-2↑; Bax↓	Kim et al. (2018)
											fragmented mitochondria↓		
	Paeonolum	*Paeonia suffruticos*	MPP^+^-treated PC12 cells	10–150 μM	24 h	MPP^+^-exposed zebrafish	100 μM	-	MMP↑; ROS↓	-	-	-	Lu et al. (2015)
	Polydatin	A non-glycosylated derivative of resveratrol	Rotenone-induced SH-SY5Y cells; Parkin shRNA transfected SH-SY5Y cells	0–500 μM	6 h	-	-	-	-	LC3-II↑, autophagic flux↑	Mfn2↑; PGC-1β↓	-	Bai et al. (2020)
	Resveratrol	*Veratrum grandiflorum* or *Polygonum cuspidatum*	Rotenone-induced SH-SY5Y cells, transfected SH-SY5Y cells; rotenone-induced PC12 cells; primary fibroblasts from two patients	0–50 μM	24/48 h	-	-	-	ATP, MMP, complex I activity, citrate synthase activity, basal oxygen consumption↑; ROS↓	LC3-II, PGC-1α, TFAM, COX 1, mtDNA/nDNA↑	Short and fragmented mitochondria↓	-	Ferretta et al. (2014), Lin et al. (2014), Lin et al. (2018), Wang et al. (2018)
	Sativex^®^ (a mixture of tetrahydrocannabinol and cannabidiol)	-	-	-	-	Transgenic mice	4.63 mg/kg of Sativex^®^	1 month	complex II and IV activity↑	-	-	-	Casarejos et al. (2013)
	α-Arbutin	*Ericaceae* species	Rotenone-induced SH-SY5Y cells	1–100 μM	6 h	Parkin-null *drosophila*	1 mM	20 days	MMP↑, ROS↓	-	-	-	Ding et al. (2020)
Terpenoids	Celastrol	*Tripterygium wilfordii*	Rotenone-induced SH-SY5Y cells	1–10 nM	24 h	-	-	-	MMP↑; ROS↓	-	-	-	Choi et al. (2014)
	Ginsenoside Re	*Panax ginseng*	PINK1 null dopaminergic cell lines	0–5 μM	-	-	-	-	Complex IV activity, NO production↑	-	-	-	Kim et al. (2012)
									LRPPRC, Hsp90, and Hsp60↑				
	Mogroside V	*Siraitia grosvenorii*	Rotenone-induced SH-SY5Y cells	25–100 μM	24 h	Rotenone-treated mice	2.5, 5 and 10 mg/kg	6 days	ATP, MMP, oxygen consumption rate↑; ROS↓	-	-	Cyt-c release, caspase 3 activity↓	Luo et al. (2022)
									SIRT3↑				
	Urolithin A	-	LPS-induced BV2 cells	2.5–10 μM	2 h	MPTP-treated mice	20 mg/kg	7 days	-	LC3-II, Parkin, PINK1 protein↑; p62↓	-	-	Qiu et al. (2022)
	Ursolic acid	*Malus domestica*, *Origanum majorana*, *Rosmarinus officinalis*,etc	-	-	-	Rotenone-treated rat	5 and 10 mg/kg	30 days	Complex I activity↑	-	COX1↑	-	Peshattiwar et al. (2020)
	Vanillic acid	-	SH-SY5Y cells	300 μM	24 h	-	-	-	-	PGC-1, TFAM↑	-	-	Ay (2022)
Glycosides	Astragalus polysaccharide	*Astragalus*	-	-	-	MPTP-induced mice	10 mg/kg	14 days	MMP↑; ROS↓	-	-	Bcl-2↑; Bax, Cyt-c release, pro-caspase-3, caspase-3↓	Liu et al. (2018a)
	Cordycepin	*Cordyceps militaris*	-	-	-	Rotenone-treated mice	2.5, 5 and 10 mg/kg	21 days	MMP, ATP, complex I activity↑; ROS↓	-	-	-	Zhang et al. (2021)
	Echinacoside	*Cistanche salsa*	6-OHDA induced PC12 cells	0.1–10 μM	24 h	-	-	-	MMP↑	-	-	--	Wang et al. (2015)
Alkaloids	Piperine and piperlonguminine	*Piper longum*	Rotenone-treated MN9D and SK-N-SH cells	250 μg/mL piperine and 5 μg/mL piperlonguminine	-	Rotenone-treated rats	12.5 and 25 mg/kg	1 week	MMP, complex I activity↑	LC3-I/II↑	-	Cyt-c release↓	Wang et al. (2016)
	Nicotine and caffeine	-	Mn^3+^ or H_2_O_2_ treated HEK293T, PC12 and SH-SY5Y cells	1–500 μM	-	-	-	-	-	Nrf2/Keap1 and PGC-1α pathway↑	-	-	Zhou et al. (2019)
Quinones	Anthraquinone	*Pleurotus ostreatus*	6-OHDA-induced SH-SY5Y cells	5–50 nM	2.5 h	-	-		MMP↑				Jin et al. (2021)
	Embelin	*Embelia ribes Burm*	N27 cells	1–5 μM	2–24 h	MPTP-induced mice	10 mg/kg	14 days	ATP, basal oxygen consumption rate↑	mtDNA, pAMPK, SIRT1, PGC1α, nuclear respiratory factor 1/2 and TFAM↑	-	-	Rao et al. (2020)
Alkenes	Isolongifolene	*Murraya koenigii*	-	-	-	Rotenone-treated rats	5, 10 and 20 mg/kg	4 weeks	-	-	-	Bcl-2↑; Bax, Cyt-c release, caspases↓	Balakrishnan et al. (2021)
Extracts	Ethanol and hexane leaf extracts	*Calyptranthes grandifolia*	6-OHDA-induced SH-SY5Y cells	300 μg/mL	3 h	-	-	-	MMP↑	-	-	caspase-3/9 activity↓	Kich et al. (2016)
	Methanol and dichloromethane extracts	*Sargassum muticum, Codium tomentosum, and Ulva compressa*	6-OHDA-induced SH-SY5Y cells	1 mg/mL	24 h	-	-	-	MMP↑	-	-	caspase-3/9 activity↓	Silva et al. (2018)
	Ethyl acetate stem bark extract	*Eucommia ulmoides*	6-OHDA-induced SH-SY5Y cells	2.5–100 μg/mL	24 h	-	-	-	MMP↑; ROS↓	-	-	Bcl-2↑; Bax, Cyt-c release and caspase-3/9↓	Kwon et al. (2014)
	Methanol fruits extract	*Zizyphus spinachristi*	MPP^+^-induced SH-SY5Y cells	20–100 μg (gallic acid equivalents)	24 h	-	-	-	MMP↑; ROS↓	-	-	Cyt-c release, caspase-3/9 activity↓	Singh et al. (2018)
	Ethanol extract of the root and rhizome	*Acanthopanax senticosus*	-	-	-	MPTP-induced mice	45.5 mg/kg	20 days	ATP, MMP↑	-	-	-	Liu et al. (2018b)
	Methanol extract	*Ganoderma lucidum*	MPP^+^-treated neuro-2a	100–800 μg/mL	0–48 h	MPTP-induced mice	400 mg/kg	4 weeks	ATP, MMP↑	-	-	Cyt-c release, caspase-3/9 activity↓	Ren et al. (2019)
	Standardized extract	*Bacopa monnieri*	Paraquat- or MPP^+^-induced SK-N-SH cells; paraquat-induced PC12 cells	12.5–100 μg/mL	1–3 h	Paraquat-treated mice	200 mg/kg	4 weeks	MMP, complex I-III activity↑; ROS↓	-	-	-	Singh et al., 2012 (2013), Hosamani et al. (2016)
	Aqueous extract of tomato seeds	-	-	-	-	Rotenone-exposed mice	50 and 100 mg/kg	3 weeks	Complex I-III activity↑	-	-	-	Gokul and Muralidhara (2014)
	Methanol extract od saffron stigmas	*Crocus sativus*	-	-	-	Rotenone-treated drosophilas	-	1 week	Complex I-III activity↑	-	-	-	Rao et al. (2016)
	Ethyl acetate fruits extract	*Morinda citrifolia*	-	-	-	Rotenone-exposed mice	150 mg/kg	1 month	Complex I and IV activity↑	-	-	Bcl-2↑; Bax, Cyt-c release, caspase-3/9 activity↓	Kishore Kumar et al. (2017)
	Standardized extract	*Centella asiatica*	-	-	-	Rotenone-exposed mice	10–100 mg/kg	20 days	Complex I↑; ROS↓	-	-	-	Teerapattarakan et al. (2018)
	Aqueous extract of red clover flowers	*Trifolium pratense*	Rotenone-exposed E17 embryos of rats	0.1–20 μg/mL	24 h	-	-	-	oxygen consumption rate↑; ROS↓	-	-	-	de Rus Jacquet et al. (2021)
	Tris-HCl extract of Ginseng total protein	*Panax ginseng*	-	-	-	Transgenic drosophilas	0.02–0.16 mg/mL	-	-	mtDNA↑	-	-	Liu et al. (2020)
	Hydroethanol extract of cocoa beans	*Theobroma cocoa*	MPP^+^-treated SH-SY5Y cells	0–8 μg/mL	20 h	-	-	-	-	PPARγ, PGC1α, Nrf2, TFAM and COX4 proteins↑	Fis1↓; Mfn2↑	Bcl-2↑	Chidambaram et al. (2020)
	Ethanol extract of leaves	*Artemisia argyi*	MPP^+^-treated SH-SY5Y cells	10–250 μg/mL	24 h	MPTP-treated mice	100 mg/kg	2 weeks	-	LC3B↑	Drp1, p-Drp1↓	-	Wu et al. (2022)
	Methanol extract of seeds	*Mucuna pruriens*	-	-	-	Transgenic drosophilas	-	0–3 months	-	-	Damaged, swollen and fragmented mitochondria↓	-	Poddighe et al. (2014)
	Aqueous extract of roots	*Decalepis hamiltonii*	-	-	-	Paraquat-treated drosophilas	0.55–2.75 mM	5 days	-	-	Fragmented mitochondrial cristae↓	-	Niveditha and Shivanandappa (2018)
	Aqueous extract of edible bird’s nest	*Aerodramus* (or *Collocalia*)	6-OHDA-induced SH-SY5Y cells	0–500 μg/mL	48 h	-	-	-	-	-	-	caspase-3/9 activity↓	Yew et al. (2014)
	Petal juicing and freeze drying	*Echium amoenum*	-	-	-	Mn^2+^ -induced mice	5 mg/kg	15 days	-	-	-	caspase-3/9 activity↓	Sadeghi et al. (2018)
	Ethanol extract of root bark	*Paeonia suffruticosa*	Embryos of rats	0.1–1 μg/mL	1 h	MPTP-treated mice	1–50 mg/kg	12 days	-	-	-	Bcl-2↑; Bax, Cyt-c release, caspase-3/9 activity↓	Kim et al. (2014)
	Methanol extract and ethanol extract	*Humulus japonicus*	6-OHDA-induced SH-SY5Y cells	0–200 μg/mL	24 h	6-OHDA-treated mice	300, 500 mg/kg	3 days	-	-	-	Cyt-c release, cleaved PARP, cleaved caspase-9 and cleaved caspase-3↓	Ryu et al. (2017)
	Hand-squeezed juice	*Citrus bergamia*	6-OHDA- or H_2_O_2_-induced SH-SY5Y cells	-	1 h	-	-	-	MMP↑; ROS↓	-	-	Bcl-2↑; Bax and p53↓	Ferlazzo et al. (2020)

### 3.1 Targeting mitochondrial respiration and bioenergetics

Mitochondria are double-layer organelles where aerobic respiration occurs. It is the major site that generates ATP via oxidative phosphorylation. The electron transfer chain, which is composed of approximately 80 polypeptides and located in the inner membrane of mitochondria, plays a vital role in ATP production. The inner membrane of mitochondria contains different transmembrane protein complexes (I-V) (Yadav et al., 2022). Along with the circulation of electrons through the entire electron transfer chain, different protein complexes set up mitochondrial membrane potential (MMP) across the inner mitochondria membrane and maintain mitochondrial integrity and perform its normal function (Papa et al., 2012). Electrons are prone to leak out of complex I and complex III in the electron transfer chain and transfer to O_2_ to produce superoxide radicals and hydrogen peroxide, which are referred as ROS (Sies et al., 2017). A balanced amount of mitochondrial ROS is involved in various beneficial processes such as various signaling pathways (Bhat et al., 2015). However, aberrant production of ROS causes severe oxidative stress and triggers PD pathogenesis via mtDNA damage, and lipid peroxidation associated with mitochondrial dysfunction (Ali et al., 2015; Badshah et al., 2016). In the respiratory chain, damage to any of the complexes also causes severe cell and mitochondrial dysfunction such as loss of MMP in PD (Qi et al., 2019). Mitochondrial respiration is an important process through which mitochondria can provide energy that supports physiological activity and body function. Many natural compounds and plant extracts can beneficially function in mitochondrial dysregulation. Most of them are flavonoids and polyphenols, in addition to glycosides, terpenoids, *etc.*

#### 3.1.1 Flavanoids

Flavanoids isolated from various sources induced mitochondrial protection and improved mitochondrial function in PD by upregulating the production of ATP, and ROS, and maintaining the mitochondrial membrane potential and activity of mitochondrial complexes. The dicarbonyl compound methylglyoxal (MG) has been shown to be linked to PD development by inducing mitochondrial dysfunction (Bakala et al., 2012). Exposure of SH-SY5Y cells to MG caused decreases in cell viability, intracellular ATP, and mitochondrial membrane potential. The flavonoid naringenin is extracted from the pericarp of *Citrus reticulata* Blanco, known as 5,7-dihydroxy-2-(4-hydroxyphenyl)-2,3-dihydrochromen-4-one. It is one of the most important polyphenolic flavanones and has been reported to have neuroprotective effects in PD possessing potent free radical scavenging properties (Lou et al., 2014). SH-SY5Y cells were pretreated for 2 h with naringenin (at 10–80 μM) and then challenged with MG at 500 μM for 24 h. Naringenin significantly increased MMP, ATP, and complex I and V activity, and attenuated the effects on mitochondrial function and the redox environment (de Oliveira et al., 2019). In another study, 80 μM Naringenin treatment in paraquat-induced SH-SY5Y cells resulted in increased cell viability, reduced oxidative stress, elevated MMP, and higher cellular ATP levels. In paraquat-induced rats, 40 mg/kg naringenin treatment resulted in significant neuroprotection against paraquat -induced behavioral deficits, oxidative stress, mitochondrial dysfunction, and astrocytosis (Ahmad et al., 2021). Calycosin, an isoflavonoid extracted from *Astragalus membranaceus*. , has been revealed to exhibit neuroprotective functions against cerebral ischemia and reperfusion-induced neurological injury, high glucose-induced oxidative stress and neuroinflammation, as well as neuronal apoptosis in earlier studies (Wang and Zhao, 2016; Hsu et al., 2020). Chaouhan et al. (2022) reported that calycosin administration increased ATP and MMP. Flies feed with 100 μM calycosin exhibited significant resistance against paraquat-induced mortality and locomotor deficits in terms of reduced oxidative stress, loss of DA neurons, depletion of dopamine content. Nobiletin is a natural polymethoxylated flavone. The disruption of mitochondrial respiratory complexes I-IV and the enhancement of ROS induced by lipopolysaccharide were ameliorated by nobiletin pretreatment, which also enhanced MMP in BV-2 microglial cells (Qi et al., 2019). Silibinin is a flavonoid extracted and isolated from the fruit of *Silybum marianum* (L.) *Gaertn* and has been widely used to exploit drugs for the treatment of diseases. Previous studies showed that silibinin could potentially exert protective effects against neuronal diseases including PD (Kujawska and Jodynis-Liebert, 2018). Silibinin restored MMP decline in mitochondrial respiration (Liu et al., 2021b). Baicalein, 5, 6, 7-trihydroxyflavone, is a flavonoid and is mainly derived from the root of the herb *Scutellaria baicalensis* Georgi, which is a well-known traditional Chinese medicine (Kuang et al., 2017). Baicalein has neuroprotective effects in PD models by exerting anti-inflammatory, anti-apoptosis, and antioxidative effects. In previous studies, it has been manifested that baicalein protected PC12 and SH-SY5Y cells against neurotoxicity induced by several toxic substances and ameliorated the neurotoxicity in rats (Mu et al., 2009; Zhang et al., 2012). It has been reported that 10 μM baicalein increased cell viability and restored mitochondrial function in SH-SY5Y cells. Baicalein administration (5 mg/kg) prevented rotenone-induced behavioral deficits, dopaminergic neuronal loss, and mitochondrial dysfunction in mice by restoring mitochondrial disorders of MMP decline. Hyperoside, which is a flavonoid glycoside, maintained MMP and ROS and improved mitochondrial function (Kwon et al., 2019). Quercetin (3,5,7,30,40-pentahydroxyflavone), found in *Bupleurum chinense* or *Crataegus pinnatifida*, has been proven to have neuroprotective and cognitive enhancing effects in different brain injury models (Selvakumar et al., 2012). A recent study reported that administration of quercetin (10 mg/kg) reduced aluminum-induced oxidative stress including decreasing ROS production and increasing mitochondrial superoxide dismutase (Sharma et al., 2016).

In addition, advanced glycation end products (AGEs) ligate to the receptor of AGEs (RAGE), improving activation of the transcription factor NF-κB, which may be involved in the development of neurodegenerative diseases (Qin et al., 2009). Myricitrin, a flavanoid contained in the root bark of *Myrica cerifera*, alleviated MG-induced dysfunction of mitochondrial bioenergetics, including an increase in ATP and MMP and a decrease in ROS at concentrations of 1 and 10 μm, and the possible mechanism is through inhibiting the AGE/RAGE/NF-κB pathway (Wang et al., 2014). Icaritin, a flavonoid extracted from *Epimedium sagittatum maxim*, reversed the decline in the levels of ATP, ADP, inosine, and citric acid in the substantia nigra of PD mice induced by MPTP. Icaritin improved the levels of SDHA, VDAC and ATP5B, which are closely related to mitochondrial respiration and energy supply (Wu et al., 2021).

#### 3.1.2 Phenols and polyphenols

In previous studies, various phenols and polyphenols exerted potent mitochondrial protection effects, such as resveratrol, mangiferin, curcumin, tea polyphenols, *etc.* Resveratrol is a natural polyphenolic compound sustained in a variety of plant species, such as *Veratrum grandiflorum* and *Polygonum cuspidatum*. It has been reported that resveratrol has potent potential for PD therapy via multiple mitochondria-related pathways (Yadav et al., 2022). The functional impacts of resveratrol on mitochondrial bioenergetics included a decrease in ROS, an increase in complex I and citrate synthase activities, basal oxygen consumption, mitochondrial ATP production, and an attenuated loss of MMP (Ferretta et al., 2014; Lin et al., 2018; Wang et al., 2018). Mangiferin is the primary polyphenol component of *Mangifera* indica L., possessing neuroprotective effects in PD. Mangiferin (20 μg/mL) pretreatment of rotenone- or MPTP-treated cells resulted in a higher MMP and significantly enhanced ATP levels in SK-N-SH neuroblastoma cells and a PD mouse model (Kavitha et al., 2014; Wang et al., 2022). Demethoxycurcumin (a natural derivative of curcumin), paeonolum and α-Arbutin, which are extracted from *Curcuma longa*, *Ericaceae* species, and Moutan cortex, respectively, have been shown to prevent MMP loss and ROS production (Lu et al., 2015; Ramkumar et al., 2017; Ding et al., 2020). In addition, Sativex^®^ (a mixture of 9-tetrahydrocannabinol and cannabidiol) significantly increased mitochondrial complex II activity and complex IV protein levels in mice (Khatri and Juvekar, 2016). Oleuropein, isolated from *Fraxinus rhynchophylla*, plays a role as a protective molecule against glutamate-induced mitochondrial dysfunction by regulating ATP and ROS levels (Kim et al., 2018). Additionally, Parkin deficiency is able to trigger mitochondrial dysfunction and dopaminergic neuronal loss (Lim and Ng, 2009). Polydatin, a nonglycosylated derivative of resveratrol extracted from grapes, protected mitochondrial function by increasing MMP and decreasing ROS in cells exposed to rotenone or knocked down Parkin (Bai et al., 2020). Putative kinase 1 (PINK1), linked to familial Parkinson’s disease, is known to affect mitochondrial function (Kim et al., 2012). Curcumin is an active natural polyphenolic compound extracted from rhizomes of *C. longa*, which is widely used. It has been reported that curcumin has multiple pharmacological effects, including the ability to inhibit the key characteristic features of PD such as ROS production, apoptosis, and cognitive deficits in cell cultures and animal models (Yang et al., 2005). In a recent study, it was shown that curcumin at different doses (50, 100, and 200 mg/kg) could significantly rescue complex II activity in mice that were given chronic administration of rotenone for 3 weeks and had significant alterations in mitochondrial enzyme complex activity (Khatri and Juvekar, 2016). The PINK1 gene plays a vital role in the maintenance and regulation of healthy mitochondria, and mutations in the PINK1 gene result in an autosomal recessive form of early-onset PD (Schapira AH et al., 1998). Subsequently, it was also reported that curcumin at a concentration of 2 μM increased MMP and spare respiratory capacity in paraquat-treated PINK1 siRNA and control SH-SY5Y cells (van der Merwe et al., 2017).

#### 3.1.3 Terpenoids

Several triterpenoids are capable of repairing the function of mitochondrial bioenergetics, including ginsenoside Re, ursolic, celastrol and mogroside V. Ginsenoside Re is one of the primary biologically active components of ginseng. Ginsenoside Re enhanced NO production and was capable of reversing the deficit in complex IV activity in PINK1 null cells by increasing LRPPRC, Hsp90, and Hsp60 levels, which are mitochondria-related complex IV assembly factors (Kim et al., 2012). Ursolic acid, a pentacyclic triterpenoid carboxylic acid, is found in many plant species. In rotenone-induced damage rats, treatment with ursolic acid at 5 and 10 mg/kg prevented inhibition of mitochondrial complex I activity in the mid-brain (Peshattiwar et al., 2020). Celastrol, a natural triterpene, protected SH-SY5Y cells from rotenone-induced MMP loss and ROS production (Choi et al., 2014). SIRT3 is present in the mitochondria and participates in multiple mitochondrial functions, including maintaining ATP levels (Ahn et al., 2008). Mogroside V, as a bioactive triterpene, recovered ROS and increased MMP, ATP production and the oxygen consumption rate in a dose-dependent manner, which may be associated with SIRT3 upregulation (Luo et al., 2022).

#### 3.1.4 Glycosides

Treatment with the phenylethanoid glycoside echinacoside significantly attenuated the MMP decrease induced by 6-hydroxydopamine (6-OHDA) in PC12 cells (Wang et al., 2015). Astragalus polysaccharide is one of the main active ingredients in astragalus. Astragalus polysaccharide maintained MMP and ROS and improved mitochondrial function (Liu H. et al., 2018). Additionally, cordycepin (3′-deoxyadenosine) is the main bioactive ingredient isolated from *Cordyceps militaris*. It effectively preserves mitochondrial function by increasing MMP, ATP content, and complex I activity and decreasing ROS levels (Zhang et al., 2021).

#### 3.1.5 Quinones

Pretreatment of cells with 50 nM anthraquinone, extracted from edible fungi *Pleurotus ostreatus*, reversed the decrease in MMP induced by 6-OHDA (Bindhu et al., 2020). Treatment with 5 μM embelin resulted in a time-dependent enhancement of the basal oxygen consumption rate and ATP production in rat N27 cells (Rao et al., 2020).

#### 3.1.6 Plant extracts

Many plant extracts restored MMP and decreased mitochondrial depolarization, including the extracts of *Calyptranthes grandifolia* leaves (Kich et al., 2016) and the seaweeds *Sargassum muticum*, *Codium tomentosum*, and *Ulva compressa* (Silva et al., 2018). The extracts of *Eucommia ulmoides*, *Zizyphus spinachristi* fruits and *Citrus bergamia* fruits restored both MMP and ROS (Kwon et al., 2014; Singh et al., 2018; Ferlazzo et al., 2020). Extracts of *Acanthopanax senticosus* and *Ganoderma lucidum* reversed mitochondrial membrane potential collapse and ATP depletion caused by MPTP (Liu S. M. et al., 2018; Ren et al., 2019). *Bacopa monnieri* L. has been proven to have neuroprotective effects, and the standardized extract also plays a role by targeting mitochondrial respiration. Pretreatment with a standardized extract of *Bacopa monnieria* maintained complex I activity in SK-N-SH cells and complexes II-III activity in the mouse striatal region and prevented MMP loss and ROS production (Singh et al., 2012, 2013; Hosamani et al., 2016). Tomato seed extracts efficiently restored ROT-induced activity loss of complexes I-II (in the hippocampus) and complexes II-III (in the striatum) (Gokul and Muralidhara, 2014). Rao et al. (2016) studied the neuroprotective efficacy of saffron methanolic extract and its bioactive constituent crocin. They both increased the activity of complexes I-III (Rao et al., 2016). Supplementation of the ethyl acetate extract of *Morinda citrifolia* significantly augmented the activity of complex I by 22% and complex IV by 23% compared to only ROT-treated rats (Kishore Kumar et al., 2017). The standardized extract of *Centella asiatica* ECa233 (30 mg/kg) protected against the inhibition of complex I and an increase in ROS (Teerapattarakan et al., 2018). *Selaginella delicatula* extract restored rotenone-induced perturbations in the activity levels of complexes I-II, MMP and activity of ATPases to normalcy among mice (Chandran and Muralidhara, 2013). *Piper longum* L. extract, containing two active alkaloids, reversed the reduction in MMP in MN9D cells caused by rotenone and protected mitochondrial complex I activity (Wang et al., 2016). Pretreatment with red clover (*Trifolium pratense*) extract and the individual isoflavone daidzein both decreased ROS levels and enhanced the oxygen consumption rate (de Rus Jacquet et al., 2021).

### 3.2 Targeting mitochondrial biogenesis and mitophagy

Mitochondrial biogenesis is a pivotal biological process that plays a critical role in maintaining mitochondrial homeostasis, and ultimately adapts to the cellular physiological demand for energy supply. Mitochondrial biogenesis is the process in which existing mitochondria divide directly to produce new mitochondria. The original mtDNA is indispensable for the mitochondrial biogenesis process due to its obligation to encode essential mitochondrial tRNAs and RNAs (Richter-Dennerlein et al., 2015). Peroxisome proliferator-activated receptor gamma coactivator (PGC)-1α is a member of a family of transcription coactivators that play a central role in the regulation of mitochondrial biogenesis and cellular energy metabolism (Finck and Kelly, 2006; Handschin and Spiegelman, 2006). PGC-1α activation is attributed to adenosine monophosphate-activated protein kinase (AMPK) induced by SIRT1 (Fernandez-Marcos and Auwerx, 2011). PGC-1α can function to activate nuclear respiratory factor (Nrf) 1/2 as a transcriptional modulator, and then bind to the mitochondrial transcription factor A (TFAM) promoter to activate TFAM and complete and promote the replication and transcription of mtDNA. Cytochrome c oxidase (MtCO-1/COX1) is a mtDNA-encoded polypeptide and is a downstream target of PGC-1α. Upregulation of COX1 transcripts provides evidence for the activation of mitochondrial biogenesis and respiratory activity (Peshattiwar et al., 2020). Therefore, the biogenesis of new mitochondria depends on the activation of SIRT1/AMPK/PGC-1α-Nrf-TFAM pathway. Plenty of studies have shown that anomalous expression of SIRT1/AMPK and the decreased expression of PGC-1α, Nrf and TFAM leaded to mitochondrial dysfunction in PD (Li et al., 2017; Mohammadipour et al., 2020).

Mitophagy is a type of selective autophagy that controls the quantity and quality of mitochondria and maintains the normal function of the mitochondrial network. Abnormal mitophagy can cause many pathological changes that can lead to Alzheimer’s disease and Parkinson’s disease (Fang X et al., 2022). Phosphatase and tensin homolog (PTEN)-induced PINK1 and Parkin, the two PD-associated genes, are involved in the selective removal of damaged mitochondria (Geisler et al., 2010). PINK1 acts upstream of Parkin in the mitochondrial quality control pathway, and the two synergistically mediate the polyubiquitination process of damaged mitochondrial surface structures or functional proteins, and play a key role in depolarizing mitophagy degradation (Matsuda et al., 2010). LC3 is the most widely used autophagosome marker to evaluate autophagic flux. During autophagy, LC3I, the cytoplasmic form, is conjugated with phosphatidylethanolamine to form LC3II, which is recruited to autophagosome membranes (Klionsky et al., 2021). During the late stages of autophagy, p62 and p62-bound polyubiquitinated proteins that are incorporated into the autophagosome are degraded in autolysosomes. Accordingly, the level of p62 indicates damaged mitochondria accumulation instead of mitophagy (Lin et al., 2014). Studies have revealed that PINK1/Parkin-dependent mitophagy augmentation occurred in treated PD models and exerted neuroprotective effects along with decreased p62 protein and increased Parkin and LC3-II levels (Kuang et al., 2017; Palikaras and Tavernarakis, 2020; Chaouhan et al., 2022).

Balance maintenance of mitochondrial biogenesis and autophagy plays a crucial role in controlling mitochondrial physiology and function (Narendra et al., 2008). Mitochondrial dysfunction of impaired mitochondrial biogenesis has been observed in neurodegenerative diseases, including Parkinson’s disease (Zhu et al., 2012). Various natural products have shown significant effects on regulating mitochondrial biogenesis and mitophagy.

#### 3.2.1 Flavanoids

The flavonoid baicalein could also increase autophagic flux in rotenone-treated mice by increasing the LC3B-II protein level (Kuang et al., 2017). PTEN-induced PINK1 and Parkin, the two PD-associated genes, are involved in the selective removal of damaged mitophagy (Geisler et al., 2010). Silibinin promoted clearance of the toxic effects of damaged mitochondria. MPTP-injected mice were protected against dopaminergic neuronal loss by oral administration of silibinin (280 mg/kg), which increased the expression of PTEN-PINK1 and Parkin, suggesting mitophagy activation (Liu et al., 2021a). The level of p62, an essential mitophagy regulator that indicates the accumulation of damaged mitochondria, was observed to be dramatically increased in paraquat-exposed flies. However, calycosin treatment inhibited this effect. It also decreased the phosphorylation levels of S6K and 4EBP1, indicating mitophagy stimulation (Chaouhan et al., 2022).

#### 3.2.2 Phenols and polyphenols

Resveratrol regulated mitochondrial biogenesis and mitophagy in PD by regulating the PGC-1α, LC3-II, p62 protein-related pathways, *etc.* Resveratrol also promoted LC3-II accumulation, inhibited p62 expression and augmented autophagic flux, which was inhibited by rotenone in SH-SY5Y cells (Lin et al., 2014; Lin et al., 2018). A significant resveratrol (25 μM) -mediated increase in the PGC-1α transcriptional activity of downstream genes TFAM and COX 1, the mtDNA/nDNA ratio, and enhanced macroautophagic flux through upregulating LC3-II levels was observed in skin fibroblasts from PD patients (Ferretta et al., 2014). Resveratrol at a concentration of 20 μM promoted LC3-II accumulation, inhibited p62 expression and augmented autophagic flux, which was inhibited by rotenone in SH-SY5Y cells (Lin et al., 2014; Lin et al., 2018). Vanillic acid is a phenolic compound found in various plants and fruits. Its treatment resulted in significant increases in the mRNA expression of PGC-1α and TFAM, and treatment with 300 µM for 24 h significantly elevated the mtDNA copy number and mitochondrial mass of SH-SY5Y cells (Ay, 2022). Urolithin A, a natural compound produced by gut bacteria from ingested ellagitannins and ellagic acid, decreased p62 protein levels and increased LC3-II and other related protein levels of Parkin and PINK1, regulating mitophagy (Qiu et al., 2022). Polydatin increased the expression of LC3-II, indicating that autophagic flux was augmented by polydatin (Bai et al., 2020).

#### 3.2.3 Terpenoids

Ursolic acid caused a prominent upregulation of COX1, the downstream gene of PGC-1α, in treated rats at a dose of 10 mg/kg, manifesting the promotion of mitochondrial biogenesis (Peshattiwar et al., 2020).

#### 3.2.4 Alkaloids

Pretreatment with the alkaloids piperine and piperlonguminine upregulated LC3-I/II in neurons compared to those without pretreatment, implying an increase in mitophagy (Wang et al., 2016). Nuclear factor erythroid 2-related factor 2-keap1 signaling pathways function to promote mitochondrial biogenesis and cell proliferation (Barone et al., 2011). The alkaloids nicotine and caffeine played a role in the activation of the nuclear factor erythroid 2-related factor 2-Keap1 and PGC-1α signaling pathways, thus regulating mitochondrial biogenesis (Zhou et al., 2019).

#### 3.2.5 Quinones

Embelin, a natural benzoquinone compound, increased mtDNA levels in a dose- and time-dependent manner in N27 cells. It also caused a substantial increase in the mitochondrial biogenesis regulators of pAMPK, SIRT1, PGC1α and mRNA levels of its downstream targets (Nrf 1/2 and TFAM) (Rao et al., 2020).

#### 3.2.6 Plant extracts

Incubation of PC12 cells with alkaloid component extracts from *Huperzia selago* and *Diphasiastrum complanatum* for 24 h evoked significant upregulation of the expression of the gene for DNA polymerase γ (Polg), which is responsible for replication of mtDNA and its repair processes (Lenkiewicz et al., 2016). Ginseng total protein administration led to an increase in mtDNA levels and supported mitochondrial biogenesis in PINK1^B9^-mutated *Drosophila* (Liu et al., 2020). *Theobroma cocoa* extract at a concentration of 10 μg/mL produced significant upregulation of PPARγ, PGC1α, Nrf2, TFAM, and COX4 proteins in 1-methyl-4-phenyl-1,2,3,6- pyridine (MPP^+^)-treated SH-SY5Y cells, mediating mitochondrial biogenesis (Chidambaram et al., 2020). Supplementation with *Artemisia argyi* extract increased LC3B expression in a dose-dependent manner in comparison with cells treated with MPP^+^ alone (Wu et al., 2022).

### 3.3 Targeting mitochondrial dynamics

Mitochondria are dynamic organelles that undergo constant fusion and fission and control mitochondrial morphology, providing energy for cells, which is vital for retaining the usual mitochondrial functions as well as cell endurance (Suarez-Rivero et al., 2016). Mitochondrial fusion supplements enzymes and mitochondrial gene products in partially damaged mitochondria to optimize mitochondrial function and reduce the accumulation of age-related mutations in the mitochondrial genome (Westermann, 2012). Fission events create multiple small mitochondria and contribute to separating the fragments of damaged mitochondria from healthy mitochondria, further facilitating their clearance by mitophagy or apoptosis when under high levels of cellular stress (Twig et al., 2008; Lin et al., 2018). Several GTPases mediate mitochondrial dynamics, such as mitochondrial fission 1 (Fis1) and dynamin-related protein-1 (Drp1) for fission in the cytoplasm and mitofusions (Mfn1 and Mfn2) and Optic Atrophy-1 (Opa1) for fusion located on the mitochondrial membrane (Zhang et al., 2021). In addition, the balance between mitochondrial fusion and fission events has been reported to be regulated by PGC-1α and 1β (Scarpulla, 2011). Unbalanced fusion and fission occur in PD with increased fragmented mitochondria or aberrant expression of related genes, and ultimately increased oxidative stress (Kim et al., 2018; Lin et al., 2018) (Chandra et al., 2019).

#### 3.3.1 Flavanoids

Silibinin administration to MPTP-treated mice restored mitochondrial dynamic disorder by decreasing Drp1 expression and increasing Mfn1 expression in the hippocampus (Liu et al., 2021b). Quercetin attenuated aluminum-induced mitochondrial swelling, loss of cristae and chromatin condensation and restored the size of mitochondria to normality in the aluminum-treated rat hippocampus, improving mitochondrial integrity and function (Sharma et al., 2016).

#### 3.3.2 Phenols and polyphenols

Resveratrol pretreatment reversed high percentages of short and fragmented mitochondria in rotenone-exposed SH-SY5Y cells (Lin et al., 2018). Oleuropein, a polyphenolic compound extracted from *F. rhynchophylla,* amended a glutamate-induced mitochondrial dynamic imbalance and reduced the number of cells with fragmented mitochondria, regulating the phosphorylation of Drp1 at amino acid residue serine 637 (Kim et al., 2018). MPTP promoted Drp1 translocation to mitochondria. However, mangiferin (10 and 40 mg/kg) markedly inhibited this effect to prevent MPTP-induced excessive mitochondrial fission with reversed expression of mitophagic proteins, including PINK1, Parkin, NIX, BNIP3, FUNDC1 and p62, and mitigated mitochondria with a disrupted and swollen structure, vague mitochondrial cristae, and condensate matrix (Wang et al., 2022).

#### 3.3.3 Plant extracts

Treatment with 0.1% standardized methanolic extract of *Mucuna pruriens* seeds significantly reduced the number of damaged, swollen and clearly fragmented mitochondria in presynaptic boutons of antennal lobes of PINK1^B9^ mutant flies compared with untreated mutants (Poddighe et al., 2014). 4-Hydroxyisophthalic acid is a bioactive component extracted from the roots of *Decalepis hamiltonii*. Fragmented mitochondrial cristae caused by paraquat exposure could be markedly reduced in the brains of flies fed 4-hydroxyisophthalic acid (Niveditha and Shivanandappa, 2018). Pretreatment with cocoa (10 μg/mL) extract downregulated the expression of mitochondrial Fis1 and upregulated the expression of Mfn2 proteins to balance mitochondrial dynamics in SH-SY5Y cells (Chidambaram et al., 2020). Artemisia leaf extract downregulated mitochondrial fission proteins (Drp1 and p-Drp1) and protected the mitochondria (Wu et al., 2022).

### 3.4 Targeting mitochondrial apoptosis

Mitochondria are crucial to the regulation of intrinsic apoptosis. In the mitochondrial apoptotic pathway, the antiapoptotic Bcl-2 and proapoptotic Bax/Bak markers have a primary role. Bax and Bak can be activated and accumulate at the outer mitochondrial membrane (Debatin et al., 2002). Then, they oligomerize and create multimeric pore complexes that alter the permeability of the outer mitochondrial membrane sufficiently, and induce leakage and overactivation of Cytochrome C (Cyt-c) (Jin et al., 2021). Cyt-c is an important mediator in the mitochondrial-associated pathway, which ultimately leads to activation of caspases (Badshah et al., 2015). Inappropriate mitochondrial apoptosis is a crucial process causing neurotoxicity in PD. Thus, a variety of natural products inhibit mitochondrial apoptosis to exert neuroprotective effects by regulating related molecules.

#### 3.4.1 Flavanoids

Baicalein inhibited the rotenone-induced increase in caspase-3 activity and significantly prevented rotenone-induced cleaved caspase-3 protein expression in mice (Kuang et al., 2017). Quercetin significantly reduced the Bax/Bcl-2 ratio and prevented the release of Cyt-c and subsequent activation of caspase-3, protecting the hippocampal region of the rat brain (Sharma et al., 2016). Pretreatment with naringenin increased the antiapoptotic protein Bcl-2 while decreasing the proapoptotic protein Bax and inhibited the MG-triggered release of Cyt-c from the mitochondria to the cytosol in SH-SY5Y cells. Meanwhile, it also significantly attenuated proapoptotic enzyme caspase-3/9 activation (de Oliveira et al., 2019). In addition, calycosin supplements alleviate paraquat -induced neurodegeneration by suppressing JNK phosphorylation and caspase-3 activation, which is responsible for DA neuronal cell death in exposed organisms (Chaouhan et al., 2022).

#### 3.4.2 Phenols and polyphenols

Glutamate treatment induced a decrease in Bcl-2 and an increase in Bax compared to control HT-22 cells. However, pretreatment with oleuropein maintained Bax/Bcl-2 expression levels in HT-22 cells exposed to glutamate treatment (Kim et al., 2018). Mangiferin prevented the increase in the expression of cytosolic Cyt-c and caspase-3/9 observed in rotenone -treated SK-N-SH cells (Kavitha et al., 2014). Green tea polyphenols increased the dysfunction of the mitochondrial apoptosis-related protein Bcl-2 and decreased Bax and caspase-3. When neurons were incubated with siBcl-2, the neuroprotective effect was abrogated (Cong et al., 2016). In rotenone-treated SH-SY5Y cells, the expression of Bax, Bad, caspase-3, caspase-6, caspase-8, and caspase-9 in mitochondria and Cyt-c in the cytosol was increased, whereas the distribution of Bcl-2, Bcl-xL and Cyt-c in mitochondria was significantly decreased. Pretreatment of cells with demethoxycurcumin gradually restored the excessive expression of these proteins (Ramkumar et al., 2017).

#### 3.4.3 Terpenoids

Caspase 3 activity and Cyt-c release into the cytosol were found to be decreased following dose-dependent treatment with mogroside V, indicating that mogroside V inhibited mitochondrial apoptosis (Luo et al., 2022).

#### 3.4.4 Glycosides

Astragalus polysaccharide played a role in MPTP-induced PD mice by increasing Bcl-2 and decreasing the expression of Bax, Cyt-c, pro-caspase-3, and caspase-3 protein significantly compared with the control group (Liu H. et al., 2018).

#### 3.4.5 Alkaloids

MN9D cells exposed to the alkaloids piperine and piperlonguminine prior to rotenone application had lower cytosolic Cyt-c levels than cells treated with rotenone only, which indicated that the apoptosis induced by rotenone was inhibited (Wang et al., 2016).

#### 3.4.6 Alkenes

Isolongifolene is a novel tricyclic sesquiterpene compound isolated from the herb *Murraya koenigii*. The biochemical effects of mitochondrial apoptosis in rotenone-treated rats were mitigated by isolongifolene (10 mg/kg), which increased the expression of anti-apoptotic Bcl-2, reduced the expression of pro-apoptotic Bax, inhibited Cyt-c release from mitochondria, and reduced the activation of caspases (Balakrishnan et al., 2021).

#### 3.4.6 Plant extracts

Plant extracts also have outstanding effects on hindering mitochondrial apoptosis. Many natural product extracts regulated apoptosis by inhibiting the activity of caspase-3/9, including the water extract of the Edible bird’s nest (Yew et al., 2014), ethanol and hexane extracts of *C. grandifolia* leaves (Kich et al., 2016), extracts of seaweeds (*S. muticum*, *C. tomentosum*, *Padina pavonica*, *U. compressa*) (Silva et al., 2018), and extracts *of Echium amoenum* petals (Sadeghi et al., 2018). *Ganoderma lucidum* extract and *Z. spinachristi* fruit extract suppressed the activation of caspase-3/9 and Cyt-c release from mitochondria into the cytoplasm (Singh et al., 2018; Ren et al., 2019). In addition, many plant extracts significantly upregulated the level of anti-apoptotic Bcl-2, downregulated the level of pro-apoptotic Bax and caspase-3/9 and blocked Cyt-c release into the cytosol, thereby alleviating neuronal loss, including the root bark extract of *Paeonia suffruticosa*, stem bark extract of *E. ulmoides*, and fruit extract of *M. citrifolia* (Kim et al., 2014; Kwon et al., 2014; Kishore Kumar et al., 2017). *Theobroma cocoa* extract increased the expression of the antiapoptotic protein Bcl-2 (Chidambaram et al., 2020). Additionally, pretreatment with the methanol extract of *Humulus japonicus* (100 and 200 μg/mL) significantly decreased the expression of cleaved PARP, cleaved caspase-9 and cleaved caspase-3. Meanwhile, Cyt-c release from the mitochondria to the cytosol was also significantly suppressed (Ryu et al., 2017). The incubation of SH-SY5Y cells with 6-OHDA significantly upregulated Bax and p53 proteins as well as downregulated Bcl-2 protein and the activity of caspase-3, which were significantly counteracted by preexposure to bergamot juice at both 0.5% and 1% concentrations (Ferlazzo et al., 2020).

### 3.5 Other effects

As the study demonstrated, *G. lucidum* extract treatment could regulate mitochondrial mobility by increasing and decreasing the speed in the anterograde and retrograde directions, respectively (Ren et al., 2019). Pretreatment of SK-N-SH cells with the alkaloids piperine and piperlonguminine blocked the opening of the mitochondrial permeability transition pore (Wang et al., 2016).

## 4 Toxicology studies of several natural products

Previous *in vitro* and clinical studies have shown that most natural compounds have no significant toxic effects within the concentration range of administration in PD (Kuang et al., 2017; Ding et al., 2020; Ahmad et al., 2021). Baicalein is not mutagenic or genotoxic and showed safety profile in preclinical and clinical toxicity studies. In a Phase I, randomized, double-blind, single-dose trial, baicalein was safe with no signs of toxicity at oral doses of 100–2,800 mg in healthy humans (Yarla et al., 2016). Silybin, silydianin, and silychristin were not cytotoxic or genotoxic at a concentration of 100 μM. Silymarin is safe in humans at therapeutic doses and is well tolerated even at a high dose of 700 mg three times a day for 24 weeks (Soleimani et al., 2019).

Natural components have high safety and serve neuroprotective roles through multiple molecular pathways. However, for this reason, when they are pharmacologically active, they exert nonspecific off-target effects on normal tissues. The toxic effects of natural products vary with the biological species and route of administration. Additionally, several natural compounds may transform into toxic substances in the process of metabolism. Therefore, side effects cannot be neglected while achieving therapeutic efficacy. Quercetin is a flavonoid that can form a semiquinone in the metabolic process, thus causing cytotoxicity (Metodiewa D et al., 1999). When several flavonoids such as naringin, and quercetin were detected, quercetin showed mutagenicity, and mutagenic activity could be detected (Bjeldanes and Chang., 1977). 945 mg/m^2^ was a safe dose of quercetin, while some patients exhibited emesis, hypertension, nephrotoxicity, and decreased serum potassium with its higher dose (Ferry DR et al., 1996; Bai et al., 2023). It has been reported that high dose curcumin induced apoptosis of normal human lymphocytes and noncancer cell lines (Li W et al., 2017; Eguchi et al., 2022). Nephrotoxic toxicity and gastrointestinal problems were detected after administration of resveratrol (Talib et al., 2022). Ursolic acid may cause liver injury at higher doses of 74, 98, and 130 mg/m^2^ with some patients exhibiting diarrhoea and elevated serum activity of aspartate aminotransferase, alanine aminotransferase and c-glu-tamyltransferase (Wang XH et al., 2013). In particular, piperine was related to decreased serum protein and increased levels of aspartate aminotransferase and alkaline phosphatase in rats, suggestive of the hepatotoxicity of piperine (Bai et al., 2023). Oral administration of anthraquinones can cause different side effects. It has been reported that a short-term toxicity of 6 weeks of 120 mg/kg oral administration was observed in female rats including disintegration, necrotic changes, and perinuclear vacuolation in the liver and kidney, which were relieved after embelin discontinuance (Poojari, 2014). When the animals received 5.44 mg/kg body weight or more of anthraquinone, acute and subchronic oral toxicity of anthraquinones including anemia and hypothyroidism, was observed in both the male and female rats (Qu et al., 2022).

## 5 Conclusion and outlook

Here, we presented studies of the neuroprotective effects of multiple natural products by targeting PD mitochondrial dysfunction. Targeting mitochondrial dysfunction is quite important for PD therapeutics, in which various kinds of natural products can play a part. Not only can pure natural products that cover diversiform structures ameliorate mitochondrial damage, but plant extracts sustaining variant components can also come into play; thus, the treatment and improvement of PD disease by natural products can be realized. As summarized above, the pharmacological mechanisms of natural products mainly include regulating mitochondrial respiration, dynamics, apoptosis, biogenesis and mitophagy. Most natural products (such as varieties of flavonoids, polyphenols, terpenoids, *etc.*) can regulate mitochondrial respiration through the production of ATP, maintenance of MMP and so on. Furthermore, in apoptosis, natural products also exert significant effects by regulating related apoptotic proteins. However, on the sides of dynamics, biogenesis and mitophagy, there are relatively few reports and in-depth investigations. In addition, it is essential to emphasize that it is difficult to illustrate the explicit structure-bioactivity relationship between natural products with structural diversity and PD established on different experimental models.

While many natural products have shown potential to treat PD, there are currently some limitations and challenges. First, many natural products can only be studied *in vitro* or in animal trials, and more clinical trials are needed to demonstrate their efficacy and safety in humans with Parkinson’s disease. In addition, the efficacy and dosage of natural products are not stable, which brings some risks to the treatment of PD. In order to overcome these limitations and challenges, future research requires more basic experimental studies to gain insight into the mechanism of action of natural products on mitochondrial dysfunction. In addition, more clinical studies are needed to evaluate the safety of natural products and their efficacy in treating human PD. Some of the best natural products can be synthesized and yield more stable, safe and controllable therapeutic doses, which will be a priority for future research.

In conclusion, natural products have great potential to be developed into new drugs for PD with exact effects on mitochondrial dysfunction. Substances of natural origins seem to be accepted more easily by patients since they are considered healthier than fully synthetic drugs. It is necessary to conduct further studies on the related mitochondrial dysfunction mechanisms of PD and other preclinical and clinical studies.
